# Letter from John M. Woodworth

**Published:** 1866-04

**Authors:** Jno. M. Woodworth

**Affiliations:** Berlin


BERLIN, February 20th, 1866.

The surgical clinics at the Chirurgisch-ophthalmologischen Klinikam der Universälät in Berlin, under direction of Prof. VON LANGENBECK, have been very rich in operations during the present winter. Of the cases brought to the operating table, carcinomatous tumors have predominated to a considerable extent. At Prof. VIRCHOW'S pathological rooms a very large proportion of the *post mortem* examinations reveal scrofulous affections of some one or all the internal organs. There is certainly a greater prevalence of scrofulous diseases among the Germans than among Americans. It seems to be confined chiefly to the poorer classes; their habits of life and mode of living probably have much to do with this. Their chief article of diet is black bread (part rye.) Meat is so dear that a poor man cannot afford any on his bill of fare, unless it be of a very poor quality. The German's diet may be said to be almost exclusively vegetable—hence, a lower state of vitality than normal exists among the people, and when disease attacks the system it readily assumes a chronic form. Chronic diseases are certainly the most prevalent, and the majority of deaths are from chronic cases. In short, to use a comparison that none but an American can fully understand, in Germany men live slow and die slow, in America they live fast and die fast. I have also noticed an almost total absence of rheumatic difficulties, and in endeavoring to account for this I very naturally attribute it to the diuretic which the German takes into his stomach every day in the form of *beer*.

The little worm, *trichina spiralis*, has caused considerable alarm among the lovers of raw pork and sausages, as well as claiming the attention of medical men of Germany. In a small city in Saxony, out of about 200 people that ate swine flesh containing trichinæ, nearly 50 per cent died. According to Prof. VIRCHOW, as soon as a trichina is liberated in the stomach it breeds hundreds of little trichinæ, which at once commence boring through the tissues in every direction and do not stop until they have reached the muscles, where they take up their abode, surrounding themselves with a kind of calcareous cocoon which is visible to the naked eye, as a minute white speck. In this condition they are believed to remain unchanged and without producing any serious trouble unless the flesh is eaten, when the same process takes place as above described. Trichinæ are killed at a temperature above 165° Fahrenheit, so there is no danger to those who eat their meat well cooked. To save the expense of a fire, the poorer classes eat their sausages and swine flesh raw. It is difficult to tell how the swine become infected with trichinæ, unless we conclude that hogs get them from the rats they eat, and if we attempt to account for the manner in which rats get trichinæ, I fear it will be necessary to come back to the starting point, and assume that rats get trichinæ from the pigs they eat!

Prof. Von Langenbeck obtains remarkably good results in resections of the joints, owing to the immobility he secures to the limb by the use of the *gypsum bandage*, which, I think, for several reasons, is preferable to the starch bandage. It is applied easier, and does not become foul from absorbing the secretions. Prof. Langenbeck's method of applying the gypsum bandage is as follows:—Placing the limb in a proper position, a flannel bandage is first applied, immediately over this a bandage of gauze or tulé, which has been previously filled with dry gypsum, is applied wet, then follows a coating of gypsum and another gauze bandage, and, lastly, a second coating of gypsum. In case of a resection or compound fracture, a bit of tin is placed over the wound before applying the flannel bandage, and after the gypsum has set a window is cut through the bandage and the tin removed. The gypsum bandage is allowed to remain until it is necessary to commence passive motion, or, in case of a fracture or resection of one of the long bones, until nature has completely restored the part. As I remarked in my last letter to you, I believe that we could have used the gypsum bandage in the field, during the last war, with the greatest advantage, and with the happiest results, in fractures and resections. I am of the opinion that the immobility it secures to the limb will allow conservative surgery to be carried as far in military as in civil practice, providing equally good food and pure air can be secured.

Prof. Langenbeck's operation for remedying the defect of cleft palate, consists in raising the periosteum in connection with the soft parts, thereby securing a bony roof to the mouth. In describing this operation I will not attempt to enter into details, except as they bear particularly on Prof. LANGENBECK'S operation. After freshening the edges of the fissure, two incisions are made on either side of the roof. The connecting mucous membrane left intact, serves to hold the flaps in proper position when they are loosened up, and also obviates the necessity of inserting ligatures, which could not be avoided if one long incision should be made instead. The incisions are made from behind forward, thereby avoiding the blood, and leaving a clear field for cutting. For this part of the operation, a strong scalpel is used, having a convex cutting edge; then by means of elevators, the periosteum is separated from the superior maxilla. For loosening up the periosteum from the palate bones, where it is not convenient to work with the elevators, a double-edged scalpel with flexed ends, and one with a broad and flexed end, are considered the best forms. When the flaps are completely loosened up, the freshened edges approximate each other, and they are then united with silk ligatures, which are inserted by means of a needle. This needle is arranged with a small sliding steel wire, after the needle is inserted this wire, which has a small hook on the end, is slipped out and the ligature secured, when the needle is withdrawn, bringing the ligature with it. Two wire hooks to insert in the mouth, connected by a rubber band to pass behind the neck, is a simple apparatus for keeping the mouth on the stretch laterally. I have witnessed six aranoplastic operations by Prof. LANGENBECK, and in all very favorable results have been secured. Ever your friend,

JNO. M. WOODWORTH.

